# Displacement Sensing for Laser Self-Mixing Interferometry by Amplitude Modulation and Integral Reconstruction

**DOI:** 10.3390/s24123785

**Published:** 2024-06-11

**Authors:** Yidan Huang, Wenzong Lai, Enguo Chen

**Affiliations:** 1 National & Local United Engineering Laboratory of Flat Panel Display Technology, College of Physics and Information Engineering, Fuzhou University, Fuzhou 350108, China; huangyd4503@163.com (Y.H.); laiwz1995@163.com (W.L.); 2 Fujian Science & Technology Innovation Laboratory for Optoelectronic Information of China, Fuzhou 350108, China

**Keywords:** self-mixing, interferometry, amplitude modulation, time–frequency spectrum

## Abstract

To robustly and adaptively reconstruct displacement, we propose the amplitude modulation integral reconstruction method (AM-IRM) for displacement sensing in a self-mixing interferometry (SMI) system. By algebraically multiplying the SMI signal with a high-frequency sinusoidal carrier, the frequency spectrum of the signal is shifted to that of the carrier. This operation overcomes the issue of frequency blurring in low-frequency signals associated with continuous wavelet transform (CWT), enabling the precise extraction of the Doppler frequency of the SMI signal. Furthermore, the synchrosqueezing wavelet transform (SSWT) is utilized to enhance the frequency resolution of the Doppler signal. Our experimental results demonstrate that the proposed method achieves a displacement reconstruction accuracy of 21.1 nm (0.89%). Additionally, our simulations demonstrated that this method can accurately reconstruct target displacement under the conditions of time-varying optical feedback intensity or a signal-to-noise ratio (SNR) of 0 dB, with a maximum root mean square (RMS) error of 22.2 nm. These results highlight its applicability in real-world environments. This method eliminates the need to manually determine the window length for time–frequency conversion, calculate the parameters of the SMI system, or add additional optical devices, making it easy to implement.

## 1. Introduction

In industrial manufacturing and biomedicine, there is a growing demand for non-contact, high-precision measurement methods. Recently, self-mixing interferometry (SMI), an optical interferometric measurement technique, has gained a significant amount of attention due to its extremely compact optical structure, self-alignment capability, and measurement accuracy comparable to traditional heterodyne interferometry [1]. SMI occurs when a laser beam is reflected back into the laser cavity by an external reflector, causing interference within the cavity. To date, SMI has been widely applied to measure various parameters, including velocity [2,3,4], displacement [5,6,7,8], angles [9,10], thickness [11,12], and absolute distance [13,14,15,16], as well as in biomedicine applications [17,18] and laser parameter assessments [19,20,21,22].

In the field of displacement measurement, various displacement reconstruction methods are employed to extract displacement signals. Among them, the fringe-counting method is renowned for its simplicity and ease of use [23]. However, this simple method has a resolution limitation of half the wavelength (λ/2). Measurement accuracy can be further enhanced through techniques such as multiple reflections [24] and even the power algorithm [25]. To further enhance the measurement accuracy, the phase unwrapping method (PUM) was introduced [26,27,28]. The PUM calculates the optical feedback phase by solving the inverse cosine of the power of the SMI signal, achieving a resolution of up to λ/40. However, this method requires the additional estimation of the motion direction. To eliminate the need for this direction estimation, researchers proposed the orthogonal phase unwrapping method (OPUM). The OPUM uses external optical devices [29,30,31,32] or additional modulation circuits [33] to generate a pair of orthogonal SMI signals. This method leverages the discontinuity of the tangent function and the orthogonal unwrapping method to calculate the optical feedback phase without requiring direction estimations. Both the PUM and OPUM require the calculation of the optical feedback strength factor *C* and the linewidth broadening factor α in the phase transcendental equation before computing the optical phase corresponding to the displacement. This process is time-consuming and complicated. Moreover, in actual measurements, the optical feedback strength factor is usually time-varying, making accurate parameter measurement challenging. To avoid the complexities and inaccuracies of parameter estimations, researchers have proposed a displacement reconstruction algorithm based on the time–frequency spectrum [34,35]. This algorithm segments the signal into overlapping, short time segments of equal length and calculates the Doppler frequency in each segment using an FFT to obtain the Doppler frequency over time. The velocity is then calculated using the Doppler frequency formula, and finally, the displacement is obtained by integration with a precision of λ/29. The major drawback of this method is that the window length of time segments must be manually adjusted for different measurement objects, preventing the implementation of automatic measurements. To address this issue, another time–frequency transformation method, the continuous wavelet transform (CWT), has been proposed. The CWT can adaptively adjust the window length according to the frequency variation in the signal, potentially resolving the problem of manual adjustment. However, the CWT suffers from frequency blurring at low frequencies, which prevents it from accurately resolving the low-frequency components of the signal, leading to reconstruction failures.

To address this shortcoming of the CWT in SMI signal extraction and to achieve more accurate displacement reconstruction, this paper innovatively proposes an amplitude modulation integration reconstruction method (AM-IRM). Specifically, a high-frequency sinusoidal carrier is algebraically multiplied with the SMI signal to shift its frequency to that of the high-frequency carrier. The synchrosqueezing wavelet transform is then applied for the time–frequency transformation, allowing for the precise extraction of the complete Doppler frequency of the signal. The target displacement is reconstructed using the Doppler velocity integration reconstruction method. This paper is divided into the following sections: (1) An introduction to the principle of the AM-IRM; (2) A simulation analysis of the impact of different carrier frequencies, time-varying *C* values, and high levels of noise interference on reconstruction accuracy; and (3) The verification of the algorithm’s feasibility through experiments involving harmonic motion and non-cooperative target vibration reconstruction.

## 2. Materials and Methods

### 2.1. The Theory of Laser SMI

**Figure 1:**
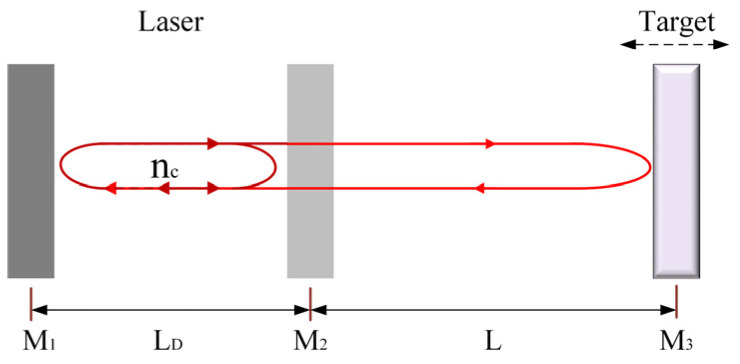
Structure diagram of three-mirror F-P cavity model of laser SMI.

The structure based on three-mirror Fabry–Pérot (F-P) cavity model is shown in Figure 1. In the diagram, $M_{1}$ and $M_{2}$ represent the two end mirrors of the laser resonator cavity with reflectivity coefficients $r_{1}$ and $r_{2}$, respectively. The length of the laser resonator cavity is denoted as $L_{D}$, and $M_{3}$ represents the surface of an external target object with a reflectivity coefficient $r_{3}$. The space between the output end of the laser and the surface of the target object forms the external cavity, with a length denoted by L.

Assuming the light wave is emitted from the left end and propagates toward the right, the initial light wave intensity is given by $Ee^{j(\omega t+\varphi_{0})}$, where E represents the initial amplitude of the light wave, ω represents the angular frequency of photons, and $\varphi_{0}$ represents the initial phase of the system. After emission, the light wave splits into two beams. One beam propagates within the resonant cavity, while the other beam transmits through the end facet $M_{2}$ of the laser and travels back and forth in the external cavity. Eventually, the two beams return to the resonant cavity and superimpose at the point $M_{1}$. When the system reaches a stable output state, the superimposed light wave should be the same as the initially emitted light wave, thus satisfying the following condition:

(1) $$Ee^{j\omega t}=r_{1}r_{2}e^{-j\omega \frac{2nL_{D}}{c}}e^{(g-\gamma )L_{D}}Ee^{j\omega t}+r_{1}(1-|r_{2}{|}^{2})r_{3}e^{-j\omega \frac{2nL_{D}+2L}{c}}e^{(g-\gamma )L_{D}}Ee^{j\omega t}$$

where $\omega 2nL_{D}/c$ represents the phase delay generated by the laser when propagating one round trip in the inner cavity, $\omega (2nL_{D}+2L)/c$ represents the phase delay generated by the laser when propagating one round trip in the outer cavity, *c* represents the speed of light in vacuum, *g* represents the gain of the laser medium, γ represents the intracavity losses of the laser, and $\xi =(1-{\left|r_{2}\right|}^{2})r_{3}/r_{2}$ represents the feedback coupling coefficient when light is reflected from an external target object back to the resonator cavity. Setting $\phi_{F}=\omega 2L/c$ and $\phi_{L_{D}}=\omega 2nL_{D}/c$, the steady-state condition of the system in the presence of external feedback light is as follows:

(2) $$1=r_{1}r_{2}e^{-j(\phi_{L_{D}}+\xi \sin\phi_{F})}e^{(g-\gamma )L_{D}+\xi \cos\phi_{F}}$$

From Equation (2), the laser gain in the presence of external feedback can be derived as follows:

(3) $$g=\gamma +\frac{1}{L_{D}}[\ln(\frac{1}{r_{1}r_{2}})-\xi \cos\phi_{F}]$$

In this case, under the influence of external light feedback, the change in laser gain can be expressed as follows:

(4) $$\Delta g=g-g_{th}=-\frac{\xi }{L_{D}}\cos\phi_{F}$$

where $g_{th}$ denotes the threshold gain of the laser. It can be observed that the change in laser gain primarily depends on the phase change associated with one round trip of the laser in the external cavity. By combining this change in threshold gain, the phase change of the system can be expressed as follows:

(5) $$\Delta \phi =2\pi \frac{2nL_{D}}{c}(v-v_{th})+\xi (\sin\phi_{F}+\alpha \cos\phi_{F})$$

where v and $v_{th}$ denote optical phase with and without optical feedback, respectively, and α represents the linewidth broadening factor of the system. Considering that the system reaches a stable output state, the phase of the system will no longer change; that is, Δϕ=0. Define the external light feedback intensity *C* as follows:

(6) $$C=\frac{\tau_{L}}{\tau_{D}}\xi \sqrt{1+\alpha^{2}}$$

where $\tau_{L}=2L/c$ and $\tau_{D}=2nL_{D}/c$ represent the time for the laser to propagate one round trip in the external cavity ($M_{2}-M_{3}$) and in the resonant cavity ($M_{1}-M_{2}$), respectively. By solving the equation Δϕ=0, the emitted frequency of the laser can be obtained as follows:

(7) $$\upsilon =\upsilon_{th}-\frac{C}{2\pi \tau_{L}}\sin(2\pi \upsilon \tau_{L}+\arctan\alpha )$$

Therefore, the phase equation for the SMI effect can be obtained as follows:

(8) $$\phi_{F}=\phi_{0}-C\sin(\phi_{F}+\arctan\alpha )$$

where $\phi_{F}$ and $\phi_{0}$ represent the output phase of the system with and without optical feedback, respectively. Under weak feedback conditions, $\phi_{F}\approx \phi_{0}$.

(9) $$\phi_{0}=\frac{4\pi L(t)}{\lambda }$$

Combined with Equation (9), the instantaneous frequency of the SMI signal can be deduced as follows:

(10) $$f_{inst}=\frac{1}{2\pi }\frac{d\phi_{0}}{dt}=\frac{2v}{\lambda }$$

As seen in Equation (10), the frequency of SMI signal is essentially Doppler frequency.

In a typical Fabry–Perot cavity model, the output optical power of the system is proportional to the carrier density, which is in turn proportional to the gain above the threshold for lasing. Consequently, the output optical power is essentially proportional to the gain above the threshold. Based on this relationship, the power equation for an SMI system can be derived as follows:

(11) $$P=P_{0}(1+k\Delta g)=P_{0}[1+k(-\frac{\xi }{L_{D}}\cos\phi_{F})]=P_{0}(1+m\cos\phi_{F})$$

where k represents a constant determined solely by the intrinsic properties of the laser, $P_{0}$ represents the laser output power when there is no optical feedback in the system, and the parameter m is the modulation coefficient of the system, which represents the visibility of the SMI signal fringes. The value of m is varies based on factors such as the strength of the optical feedback, the distance between the laser and the external target, and the reflectivity of the target surface. A higher m value indicates more pronounced SMI fringes, thereby potentially enhancing sensitivity and resolution of SMI applications.

### 2.2. Amplitude Modulation Combined with SSWT for Displacement Measurement

The basis of the SSWT is the CWT, and the CWT can be expressed as follows:

(12) $$W(a,b)=\frac{1}{\sqrt{a}}\int_{-\infty }^{\infty }\Delta P(t)\bar{\psi (\frac{t-b}{a})}dt$$

where a is the scale factor, which is inversely proportional to the frequency, and b is the translation factor, which is time dependent. ψ(t) is the wavelet basis function. W(a,b) reveals how similar the signal is to the wavelet basis function at (a,b).

**Figure 2:**
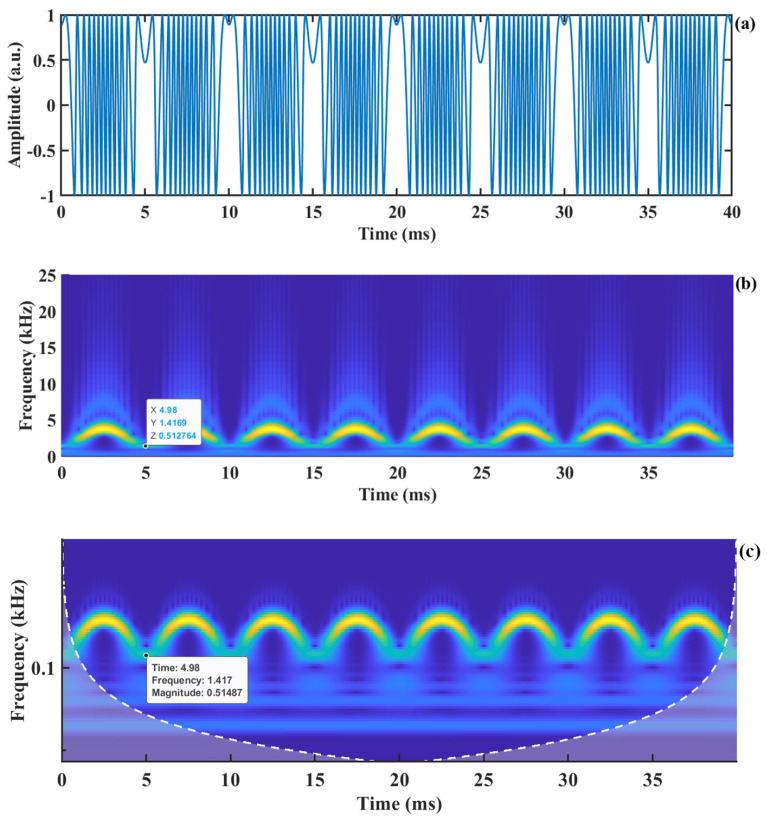
Time−frequency diagram of SMI signal from CWT: (**a**) SMI signal, (**b**) time−frequency spectrum, and (**c**) the logarithmic spectrum of (**b**).

**Figure 3:**
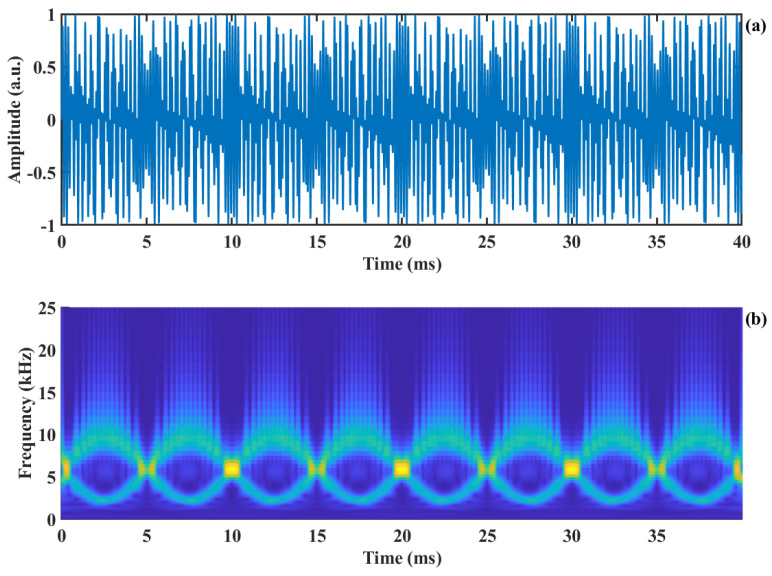
Effect of amplitude modulation on signal CWT transformation: (**a**) AM-SMI signal, (**b**) the corresponding CWT time−frequency representation of (**a**).

Figure 2a shows the SMI signal corresponding to a displacement with an amplitude of 2 µm and a frequency of 100 Hz. By performing CWT on this signal, we construct Figure 2b. To observe the frequency values in the low-frequency region more clearly, the vertical axis is transformed to a logarithmic scale, resulting in Figure 2c. From Figure 2c, it is evident that in the time–frequency spectrum, the signal with a frequency below 1.4 kHz becomes blurred, causing the main frequency in the low-frequency region to be overwhelmed. When the instantaneous velocity of the motion is relatively slow, the main frequency of the generated Doppler signal becomes very small. This low-frequency signal becomes challenging to detect in the time–frequency spectrum obtained through CWT, ultimately failing to detect the velocity of the object. To address the frequency resolution issue of CWT in the low-frequency region, we employ a high-frequency sinusoidal carrier signal $f_{shift}$ to multiply the SMI signal, resulting in an amplitude-modulated SMI (AM-SMI) signal, as shown in Figure 3a. This operation shifts the spectrum of the SMI signal to the carrier frequency region, as shown in Figure 3b, where the carrier frequency is 6 kHz. It can be observed that in the time–frequency spectrum, the signal energy above the carrier frequency leaks toward higher frequencies, causing the signal energy to be dispersed [36], which is not conducive to the extraction of the main frequency ridge. To suppress the spectral leakage problem of CWT and enhance the spectral resolution, SSWT is introduced to compress the frequency values within the time–frequency grid of the signal, which makes the frequency values of the signal more concentrated and improves the frequency resolution at the same time.

In the synchronous squeeze period, energy is transferred from the time-scale plane ω(a,b) to the time–frequency plane (ω(a,b),b). The synchronous squeeze value $T(\omega_{l},b)$ of the wavelet transform can be obtained by compressing the values of the interval $\left[\omega_{l}-\Delta \omega /2,\;\omega_{l}+\Delta \omega /2\right]$ around any central frequency $\;\omega_{l}\;$ as shown below:

(13) $$T(\omega_{l},b)=\int_{A(b)}W(a,b)a_{i}^{-3/2}da\;\;A(b)=\{a,W(a,b)\neq 0\}$$

**Figure 4:**
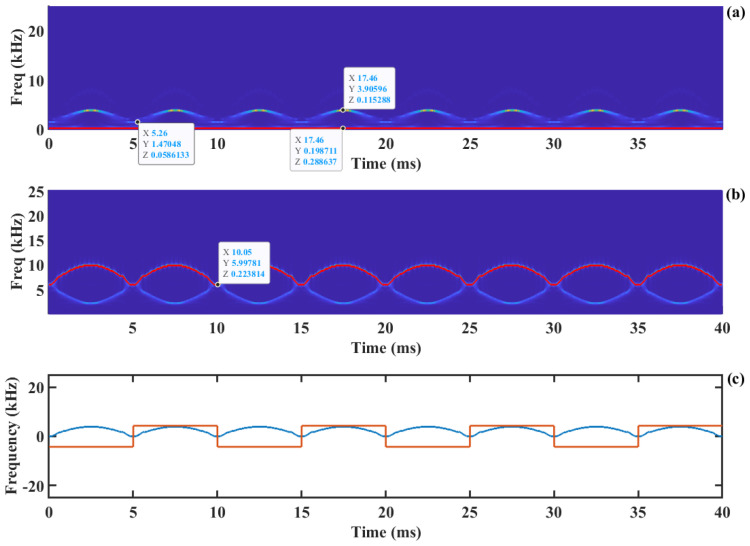
The extraction of time−frequency ridge: (**a**) SSWT time−frequency representation without amplitude modulation, (**b**) SSWT time−frequency representation of the AM-SMI signal, (**c**) Extracted time−frequency ridge and direction information.

In the time–frequency diagram $\left|T(\omega_{l},b)\right|$, the frequency with the largest energy is called the time–frequency ridge, which is expressed through Equation (14) and shown in the red line of Figure 4b as follows:

(14) $$f_{\max}=\;2\pi *\max(T(\omega_{l},b))$$

Meanwhile, $f_{\max}=\;f_{D}\;+f_{shift}$, where $f_{D}$ is the Doppler frequency corresponding to the target motion. Compared to Figure 3b, the energy of the time–frequency ridge (the red line in Figure 4b) of the signal is more concentrated, and the frequency resolution is improved. As shown in Figure 4b, the low-frequency component of the SMI signal is shifted to the carrier frequency, which can be clearly distinguished as indicated by the *Y*-axis label in the legend. Then, the absolute value of the Doppler velocity is obtained by subtracting the carrier frequency $f_{shift}$ from the time–frequency ridge, as described in Equation (15) and shown by the blue line in Figure 4c, which is referred to as frequency down-conversion. The time–frequency spectrum from SSWT is shown without amplitude modulation in Figure 4a. It is evident that the DC component replaces the Doppler signal as the time–frequency ridge, which contributes to the Doppler signal extraction failure.

(15) $$f_{D}=\left|f_{\max}-f_{shift}\right|$$

Furthermore, the direction of velocity is calculated using the method described in the literature [37], which is shown as the red line in Figure 4c. In this figure, the high level of the square wave indicates a positive velocity direction, signifying that the object moves away from the laser. Conversely, the low level of the square wave indicates a negative velocity direction, indicating that the object moves toward the laser.

**Figure 5:**
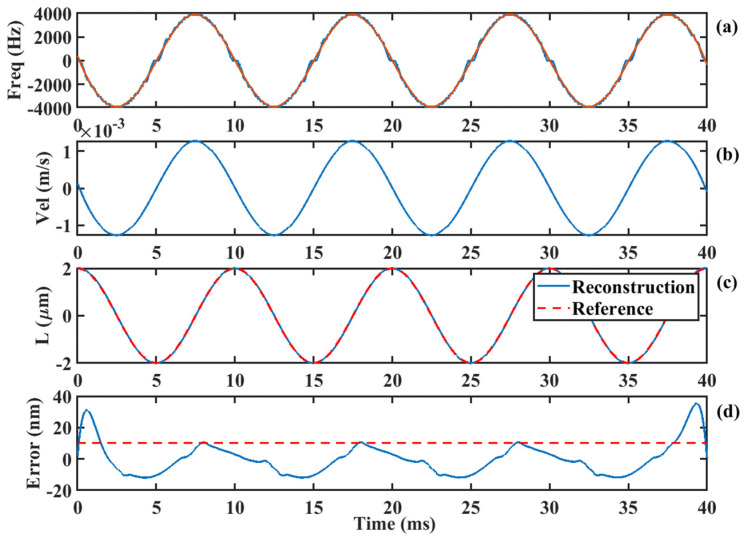
Displacement reconstruction process based on time−frequency ridge line: (**a**) Doppler frequency, (**b**) Doppler velocity, (**c**) reconstructed displacement and its reference, (**d**) error curve.

Based on the motion direction information, Doppler frequency recovery and displacement reconstruction are performed, as illustrated in Figure 5. The blue line in Figure 5a represents the Doppler signal corresponding to the target motion. It can be observed that the frequency curve exhibits discontinuous jumps near the zero frequency, which occurs because the frequency variation reaches its minimum at the zero frequency, and the frequency resolution of the time–frequency spectrum is insufficient at this point.

We employ the generalized regression neural network (GRNN) mentioned in the literature [34] to fit this discontinuous variation. The fitted Doppler curve is shown as the red line in Figure 5a. The relationship between the Doppler frequency and velocity is as follows:

(16) $$f_{D}=\frac{2v}{\lambda }$$

**Figure 6:**
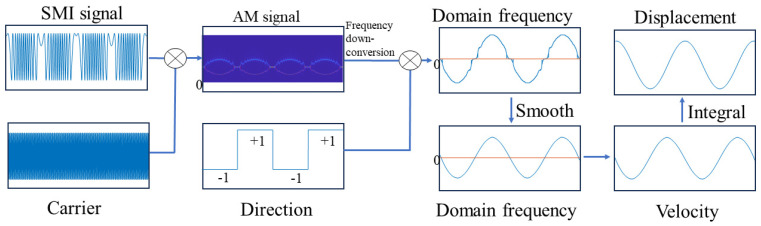
Flow chart of the proposed method.

The target motion velocity can be obtained from the fitted Doppler frequency curve. Subsequently, by integrating the velocity, the displacement curve of the target can be acquired, as shown by the blue line in Figure 5c, with the red line representing the reference displacement curve. The reconstruction error of the displacement is shown in Figure 5d, and the RMS error is 10.1 nm, as indicated by the red dashed line. The overall algorithm flow chart is illustrated in Figure 6.

## 3. Results

### 3.1. Simulated Results

The theory of the SSWT used in displacement measurement is described in the previous section. On this basis, a series of simulations are carried out to demonstrate its performance. The parameters used in the simulations are listed in Table 1.

**Figure 7:**
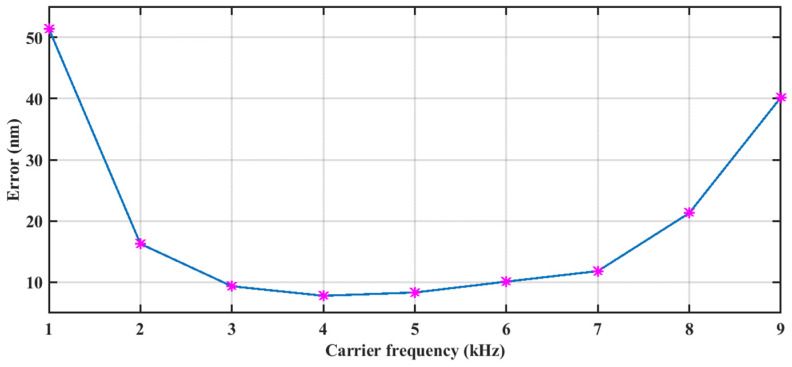
The displacement reconstruction error of different carrier frequencies.

The carrier frequency influences the extraction of the Doppler frequency, which in turn affects the accuracy of the displacement reconstruction. To investigate this, we simulated the impact of different carrier frequencies on the signal reconstruction accuracy, and the results are shown in Figure 7. It can be observed that an optimal modulation frequency exists around the carrier frequency, with the most suitable range being 3 kHz to 6 kHz, and the minimum RMS error is 7.8 nm.

It is demonstrated that when we shift the entire frequency curve out of the low-frequency blurred region, the time–frequency ridge of the signal can be completely extracted. For convenience, the time–frequency ridge can be accurately extracted when the carrier frequency satisfies the following condition:

(17) $$f_{shift}>\max(f_{inst})$$

where $f_{inst}$ is the instantaneous frequency of the SMI signal. When combined with Equation (9), $f_{inst}$ can be expressed as follows:

(18) $$f_{inst}=\frac{1}{2\pi }\frac{d\phi_{F}}{dt}\approx \frac{1}{2\pi }\frac{d}{dt}\frac{4\pi L(t)}{\lambda }=\frac{4\pi A_{0}\sin(2\pi f_{0}t)}{\lambda }$$

By combining Equation (17) and Equation (18), the following relation is derived:

(19) $$f_{shift}>\frac{4\pi A_{0}f_{0}}{\lambda }$$

On the other hand, when the carrier frequency is too high, the level of frequency leakage will become more severe, so the minimum carrier frequency can be selected.

**Figure 8:**
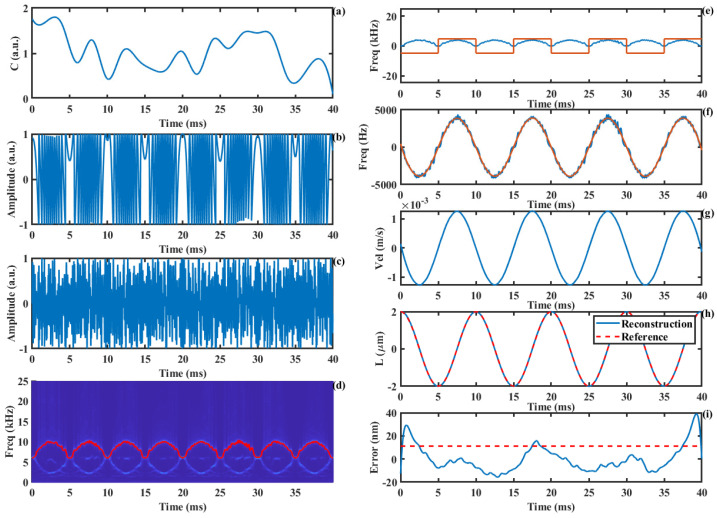
The displacement reconstruction process under the condition of variable *C* value: (**a**) random *C* value, (**b**) SMI signal, (**c**) AM-SMI signal, (**d**) time−frequency ridge, (**e**) absolute value of Doppler frequency and the direction information, (**f**) Doppler frequency, (**g**) Doppler velocity, (**h**) displacement, (**i**) error curve.

In practical measurements, the movement of the laser focal point on the object’s surface causes changes in the geometric region illuminated by the light spot, leading to variations in the *C* value of the SMI system. Compared to the PUM, our approach demonstrates a more stable displacement reconstruction accuracy under conditions of time-varying *C* values. Figure 8a shows the simulated *C* value with random variations in the range of 0.1 to 1.8. Figure 8b presents the corresponding SMI signal, while Figure 8c depicts the amplitude-modulated SMI signal. Performing the SSWT on the modulated SMI signal yields the time–frequency plot shown in Figure 8d. It can be observed that the time–frequency curve is symmetric around the carrier frequency, and the frequency of the signal is fully displayed when Equation (17) is satisfied. By extracting the maximum frequency value at each time instant, the time–frequency ridge is obtained, as indicated by the red line. Figure 8e shows the extracted non-directional Doppler frequency curve and direction information. Using these two components, the true time-varying Doppler frequency information is calculated, as shown by the blue line in Figure 8f, with its smoothed curve represented by the red line. Based on the relationship between the Doppler frequency and velocity, the motion velocity is calculated, as shown in Figure 8g. Subsequently, the target displacement is calculated through integration, as shown in Figure 8h. The reconstructed displacement is shown in Figure 8i, with a root mean square (RMS) error of 11.1 nm.

**Figure 9:**
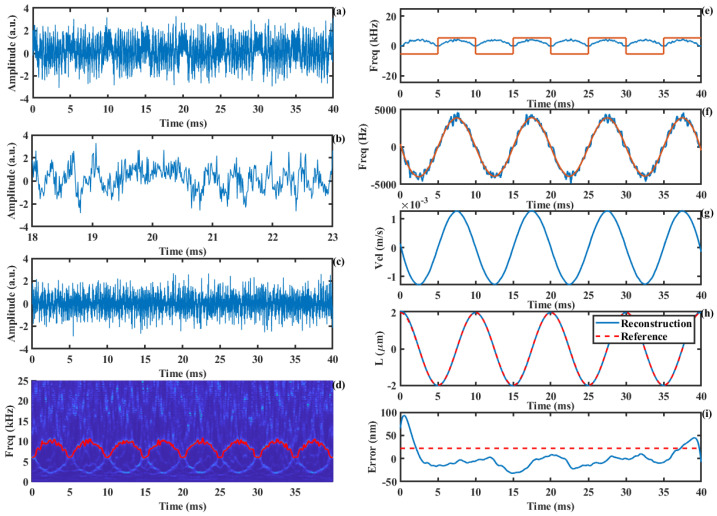
The displacement reconstruction accuracy under the condition of SNR = 0 dB: (**a**) SMI signal, (**b**) details of (**a**), (**c**) AM-SMI signal, (**d**) time−frequency ridge, (**e**) absolute value of Doppler frequency and the direction information, (**f**) Doppler frequency, (**g**) Doppler velocity, (**h**) displacement, (**i**) error curve.

The proposed method not only effectively resists the influence of time-varying *C* values but also exhibits strong noise immunity. Even under 0 dB SNR conditions, the algorithm maintains a high displacement reconstruction accuracy, as shown in Figure 9. Figure 9a presents the SMI signal with an SNR of 0 dB, while Figure 9b shows a locally magnified portion of the signal, revealing that the signal details are almost lost at this point. Figure 9c illustrates the AM-SMI signal. As shown in Figure 9d, even under strong noise interference, the time–frequency ridge of the signal can still be effectively extracted. The displacement reconstruction process based on the time–frequency ridge is the same as described earlier. The reconstruction error curve is shown in Figure 9i, with an RMS error of 22.2 nm.

### 3.2. Experiment Results

**Figure 10:**
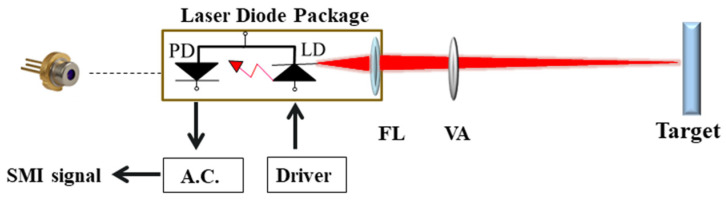
Experimental setup.

To verify the feasibility of this method, we set up an experimental system as shown in Figure 10. The laser source used is a multi-longitudinal-mode semiconductor laser diode (LD650P007, Thorlabs (Newton, NJ, USA)) with a wavelength of 650 nm and an output power of 7 mW. The laser diode (LD) is integrated with a photodetector (PD) in a cylindrical metal package. An adjustable focus lens (FL) housed on the front of the package focuses the laser on the target. A variable attenuator (VA) placed in the external cavity is used to adjust the optical feedback factor [38]. The target is placed 30 cm away from the laser, and a small amount of light is reflected or scattered back into the laser cavity. The optical intensity of the SMI signal is converted to the current, converted to the voltage, amplified, and filtered by the analog circuit (A.C.). Finally, the waveform of the SMI signal is observed and collected by an oscilloscope (TBS2000B SERIES, Tektronix, Beaverton, OR, USA), and the calculation is processed on a computer. The sampling frequency of the oscilloscope is set to 100 kHz. The measured object in the experiment is a loudspeaker with a driven frequency of 100 Hz.

**Figure 11:**
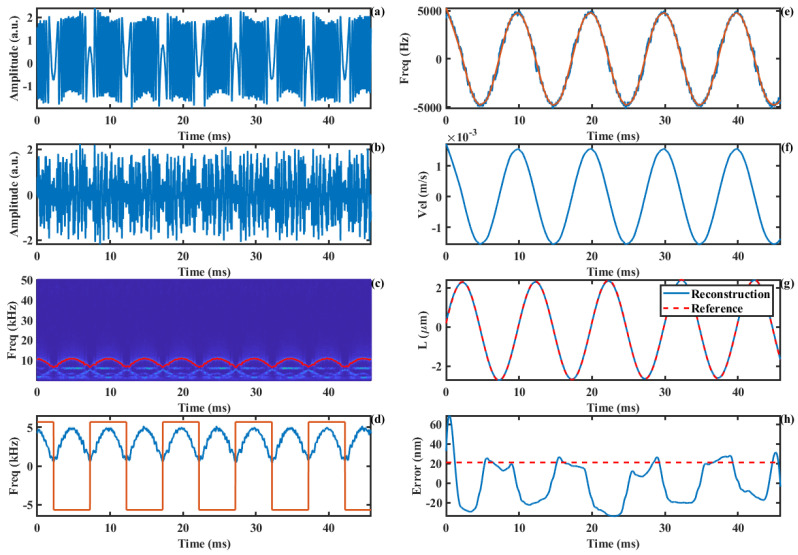
Harmonic displacement reconstruction experiment: (**a**) SMI signal, (**b**) AM-SMI signal, (**c**) time−frequency ridge, (**d**) absolute value of Doppler frequency and the direction information, (**e**) Doppler frequency, (**f**) Doppler velocity, (**g**) displacement, (**h**) error curve.

The experimentally acquired SMI signal is shown in Figure 11a. We set the carrier frequency to 6 kHz, and the corresponding AM-SMI signal is shown in Figure 11b. At this carrier frequency, the maximum value of the signal is approximately equal to the carrier frequency, resulting in a relatively clear time–frequency ridge. The extracted time–frequency ridge and direction information are presented in Figure 11d. The true Doppler frequency curve that was recovered based on the direction information is shown by the blue line in Figure 11e, while the smoothed curve is represented by the red line. The motion velocity curve of the loudspeaker, obtained by the Doppler frequency shift formula, is illustrated in Figure 11f. Furthermore, the displacement curve obtained through velocity integration is shown by the blue line in Figure 11g, with the red line indicating the reference displacement. Compared to the reference displacement, the measurement error of our proposed method is presented in Figure 11h, with an RMS measurement error of 21.1 nm.

**Figure 12:**
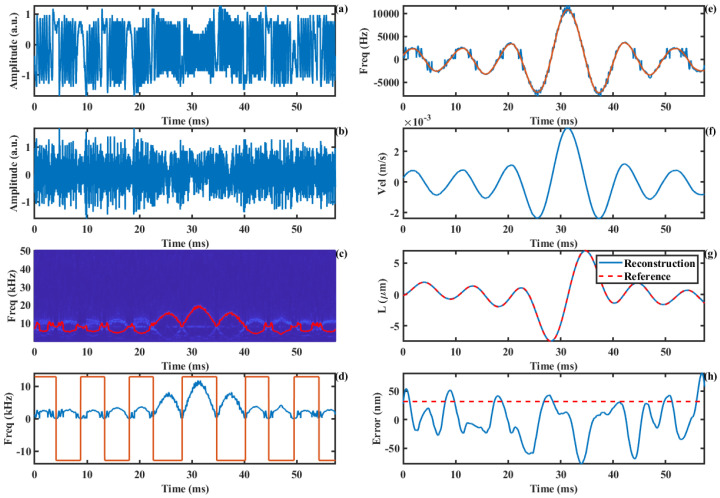
Displacement reconstruction experiment of non−cooperative target: (**a**) SMI signal, (**b**) AM-SMI signal, (**c**) time−frequency ridge, (**d**) absolute value of Doppler frequency and the direction information, (**e**) Doppler frequency, (**f**) Doppler velocity, (**g**) displacement, (**h**) error curve.

Furthermore, we employ an arbitrary waveform generator (MHS2300A-02M, Junctek, Zhengzhou, China) to drive the loudspeaker, using a carrier frequency of 10 kHz. The obtained SMI signal is shown in Figure 12a. The resulting AM-SMI signal is shown in Figure 12b, and the corresponding SSWT time–frequency plot is presented in Figure 12c, with the time–frequency ridge marked by a red line. The extracted time–frequency ridge and direction information are displayed in Figure 12d. The recovered true Doppler signal and its smoothed curve are shown in Figure 12e. The corresponding velocity curve and displacement reconstruction curve are illustrated in Figure 12g. The error curve is provided in Figure 12h, showing an RMS error of 31.9 nm.

## 4. Discussion

(1) The modulation frequency can also be smaller than the $f_{\max}$, as long as the signal is shifted out of the low-frequency blurred region. This is because we calculate the absolute value of the difference between the time–frequency ridge and the carrier frequency. Regardless of which is larger, we can obtain the value of the Doppler frequency as described in Equation (15). In other words, if the signal frequency below the carrier frequency becomes blurred, it can be replaced by the symmetric frequency signal above the carrier frequency, which has a minimal impact on the extraction of the Doppler frequency. As shown in Figure 12e, the extracted maximum signal frequency is approximately 12 kHz, which is higher than the carrier frequency. However, this does not affect the accurate extraction of the Doppler frequency curve of the signal.
(2) This algorithm employs the GRNN fitting method to smooth the non-smooth Doppler frequency curve around the zero frequency. In the error curve, it can be observed that the error reaches an extreme value at the point at which the velocity is zero, corresponding to the changes in the displacement direction. Consequently, for motion involving multiple frequency components and complex velocity curves, the reconstruction error tends to increase.
(3) In actual measurements, speckle interference is likely to occur in the SMI system. According to the description in the literature [37], the gain of SMI signals generated at different points on the surface of an object can be expressed as follows:
  (20) $$\Delta G(x,y)=-\frac{\beta (x,y)}{2L_{D}}\cos[\omega \tau +\phi (x,y)]$$
  where $L_{D}$ is the length of the internal cavity, ω is the optical frequency with feedback, and τ is the time that light propagates in the external cavity. $\beta (x,y)=KU_{0}(x,y)$, where K is an intracavity coupling coefficient and is constant when the laser is selected. $U_{0}(x,y)$ and ϕ(x,y) represent the changes in the amplitude and phase of the electric field, which are generated by target surface roughness, and x and y are coordinates in the coordinate planes of the target surface. From Equation (20), it is apparent that the signal intensity is closely linked to the geometrical shape and reflectivity of the target surface. Different points on the reflection surface produce different SMI signals, and the amplitude of the final detected signal is modulated by the roughness and reflectivity of the target surface in the light spot. When the target moves relative to the light spot, the area of the target surface covered by the light spot changes, which leads to a change in the signal gain at that moment. Consequently, when the target moves, an envelope appears on the SMI signal, whose amplitude is related to the roughness of the object’s surface and the motion amplitude. Moreover, the larger the amplitude, the more pronounced the speckle envelope, as demonstrated by the comparison between Figure 11a and Figure 12a. In practical measurements, the surface roughness of different objects varies considerably, necessitating an algorithm with strong speckle interference suppression capabilities. From an algorithmic perspective, our algorithm exhibits an excellent level of resilience against speckle interference. This is because changes in the signal amplitude do not affect the determination of the dominant frequency at any given moment. Only when the speckle is large enough to cause signal baseline drift, resulting in the DC component becoming the dominant frequency, will it impact the reconstruction accuracy.

## 5. Conclusions

In conclusion, this study proposed the AM-IRM to reconstruct the displacement in SMI system. By innovatively shifting the signal frequency to a higher-frequency region through algebraic multiplication with a high-frequency carrier, we obtain the time–frequency spectrum of the signal using the SSWT. The Doppler frequency of target motion is extracted by identifying the time–frequency ridge and subsequently subtracting the carrier frequency. Then the displacement of the target motion is reconstructed using the Doppler velocity integration method. The reconstruction accuracy under conditions of time-varying *C* values and a low signal-to-noise ratio in practical scenarios is analyzed through simulations, demonstrating the strong robustness of the proposed method. Experimentally, the displacement reconstruction accuracy for harmonic vibration reaches 21.1 nm (0.89%). This method overcomes the critical issue of our inability to extract low-frequency components of signals in wavelet transforms. Furthermore, by employing the synchrosqueezing wavelet transform, the frequency resolution in the time–frequency spectrum is enhanced, the spectral leakage problem of high-frequency components in wavelet transforms is mitigated, and the accuracy of the Doppler frequency curve is effectively improved. This study provides a practical and stable solution for displacement reconstruction methods based on SMI signals.

## Figures and Tables

**Table 1:** Simulation Parameters’ Setup.

Parameters	Value
Feedback Level Factor (C)	0.5
Line-width Enhancement Factor (α)	4
Sampling Points (*N*)	4000
$\mathrm{Sampling}\;\mathrm{Frequency}\;(f_{s})$	100 kHz
$\mathrm{Target}\;\mathrm{Vibration}\;\mathrm{Frequency}\;(f_{0})$	100 Hz
$\mathrm{Target}\;\mathrm{Vibration}\;\mathrm{Amplitude}\;(A_{0})$	2 μm
$\mathrm{Wavelength}\;(\lambda_{0})$	650 nm
$\mathrm{Carrier}\;\mathrm{Frequency}\;(f_{shift})$	6 kHz

## Data Availability

Data are contained within the article.
